# Fast Electrochemical Actuator with Ti Electrodes in the Current Stabilization Regime

**DOI:** 10.3390/mi13020283

**Published:** 2022-02-10

**Authors:** Ilia V. Uvarov, Artem E. Melenev, Vitaly B. Svetovoy

**Affiliations:** 1 Valiev Institute of Physics and Technology of Russian Academy of Sciences, Yaroslavl Branch, Universitetskaya 21, 150007 Yaroslavl, Russia; i.v.uvarov@bk.ru (I.V.U.); timon_man@mail.ru (A.E.M.); 2 Frumkin Institute of Physical Chemistry and Electrochemistry, Russian Academy of Sciences, Leninsky Prospect 31 Bld. 4, 119071 Moscow, Russia

**Keywords:** electrochemical actuators, nanobubbles, water electrolysis, microfluidics, MEMS

## Abstract

The actuators needed for autonomous microfluidic devices have to be compact, low-power-consuming, and compatible with microtechnology. The electrochemical actuators could be good candidates, but they suffer from a long response time due to slow gas termination. An actuator in which the gas is terminated orders of magnitude faster has been demonstrated recently. It uses water electrolysis performed by short voltage pulses of alternating polarity (AP). However, oxidation of Ti electrodes leads to a rapid decrease in the performance. In this paper, we demonstrate a special driving regime of the actuator, which is able to support a constant stroke for at least 10${}^{5}$ cycles. The result is achieved using a new driving regime when a series of AP pulses are interspersed with a series of single-polarity (SP) pulses. The new regime is realized by a special pulse generator that automatically adjusts the amplitude of the SP pulses to keep the current flowing through the electrodes at a fixed level. The SP pulses increase the power consumption by 15–60% compared to the normal AP operation and make the membrane oscillate in a slightly lifted position.

## 1. Introduction

Microfluidic systems are widely used in biology, chemistry, and medicine for cell culturing and manipulation [1,2], synthesis of materials [3,4], pathogen detection [5,6], and other purposes. One of the recent advances in microfluidics is implantable drug delivery modules, which provide controlled release of the drug directly to the target organ or tissue, bypassing the physiological barriers of the body [7,8,9,10]. These modules require a built-in pump that pushes the liquid through the microneedle. The pump has to be small, consume low power, and be compatible with microtechnology. The pumping mechanisms known to date include piezoelectric [11,12,13,14,15], electrostatic [16,17,18,19,20], electromagnetic [21,22,23,24,25], and thermopneumatic [26,27,28,29,30] principles. However, each of them has inherent drawbacks. A promising alternative is an electrochemical actuator [31,32,33,34,35,36,37], which consists of a working chamber with two electrodes inside. The chamber is covered by a flexible membrane and filled with an aqueous electrolyte solution. A constant voltage applied to the electrodes induces the electrochemical process. Hydrogen and oxygen bubbles grow above the cathode and anode, respectively, and push the membrane up. When the voltage is turned off, the gases recombine back into water and the membrane returns to its initial position. The reciprocating movement of the membrane pumps a fluid in the direction restricted by the valves. The electrochemical actuator develops large force and stroke in combination with the low power consumption and heat generation. A simple design makes the device easy to fabricate and integrate into microfluidic systems. The main disadvantage is the long response time, which is typically several minutes due to the slow recombination of the gases [36,37]. This leads to a rather low operating frequency of around 0.01 Hz.

A new principle of electrochemical actuation has been proven to be feasible [38], and, recently, a realistic actuator has been demonstrated and tested [39]. It works several orders of magnitude faster than the devices using DC electrolysis. A series of alternating-polarity (AP) pulses is applied to one electrode, while the other one is grounded. Due to change in the polarity, the bubbles containing H${}_{2}$ and O${}_{2}$ gases are produced above both electrodes. A short duration of pulses (1–5 μs) limits the bubble size by a value of around 100 nm [40]. Overpressure is created in the chamber and pushes the membrane up. After switching off the pulses, the bubbles disappear in milliseconds due to a spontaneous combustion reaction between hydrogen and oxygen in a nanobubble (see a review [41]). As a result, the device can operate at frequencies as high as 1 kHz.

The fast actuator has, however, limited performance due to the rapid degradation of the electrodes. The reason is that the AP process is characterized by an extremely high current density ∼100 A/cm${}^{2}$, which quickly destroys the platinum or gold electrodes [42] typically used in DC actuators. Among many metals, titanium is the most resistant to damage, although it is prone to oxidation, which reduces the current flowing through the electrochemical cell [43]. For Ti electrodes, the reduction of the current with time is the main obstacle to using the actuator for pumping purposes.

A new regime to drive the actuator has been proposed [44] for which the idle intervals between the series of AP pulses are filled with single-polarity (SP) pulses. Preliminary experiments [44] demonstrated that this idea could improve the long-term performance of the actuator, but the amplitude of the SP pulses requires fast adjustment to stabilize the stroke. In this paper, we demonstrate, for the first time the operation of the actuator during around 10${}^{5}$ cycles without a significant loss of performance. We analyze the stabilization process, power consumption, and electrode oxidation in the new regime.

## 2. Materials and Methods

**Figure 1:**
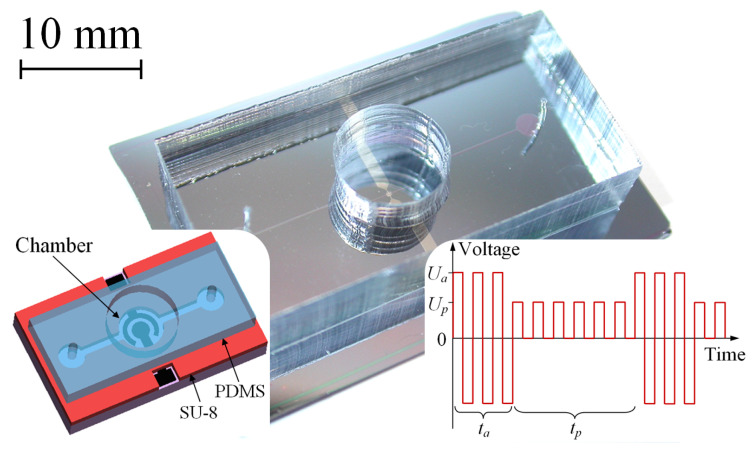
A photo of the fast electrochemical actuator. The left-hand inset shows a schematic view of the device, while the right-hand one demonstrates the driving signal applied to the actuator in the new working regime.

The actuator is shown in Figure 1. The chip has lateral sizes of 35×20 mm and its height is 4 mm. The working chamber, with a diameter of 500μm, is located in the center of the chip and contains two electrodes deposited on the oxidized silicon wafer. The bilayer electrodes are made of aluminum (bottom, conducting) and titanium (top, working) layers with a thickness of 0.5μm each. The electrodes have a concentric shape that provides the largest deflection of the membrane compared to other designs [44]. The side walls of the chamber are made of a SU-8 photoresist with a thickness of 16μm. The chamber is sealed with a 30μm thick PDMS membrane and is filled with the electrolyte through 100μm wide channels formed in the SU-8 layer. A molar solution of Na${}_{2}$SO${}_{4}$ in distilled water is used as the electrolyte.

The actuator is driven by a series of rectangular AP voltage pulses applied to one of the electrodes, while the other one is grounded. Each series consists of *N* = 20,000 pulses with the amplitude $U_{a}=12$ V and has the duration (active time) $t_{a}=N/2f=20$ ms, where f=500 kHz is the frequency of pulses. The time interval between the series (passive time) is $t_{p}=80$ ms. Thus, the working cycle lasts 100 ms, and the operating frequency is $f_{c}={\left(t_{a}+t_{p}\right)}^{-1}=10$ Hz. During the active time, the nanobubbles containing hydrogen and those containing oxygen are generated above both electrodes and push the membrane upward. During the passive time, the gases recombine back into water, and the membrane returns to the initial position. The actuator can operate at frequencies $f_{c}$ up to 1 kHz [39], but here we use a typical value $f_{c}=10$ Hz for which the gas is completely terminated for each cycle.

In the normal operation regime, the voltage is not applied to the electrodes during the passive time. The performance of the actuator is dramatically reduced in minutes due to the oxidation of the titanium coating [44]. To avoid the reduction of the stroke, it was proposed [44] to fill the passive time with SP pulses, as shown in the right-hand inset of Figure 1. The polarity of theses pulses can be either positive or negative; their amplitude is $U_{p}$ but the frequency is the same as for the AP pulses. It was assumed [44] that a series of SP pulses reduces the Ti oxide, increasing the current flowing through the electrodes during the subsequent AP series. A larger current produces more gas, increasing the membrane deflection.

To support such a new regime, one has to keep the amplitude of the SP pulses near a certain threshold. Above the threshold, the concentration of nanobubbles becomes too large, resulting in an uncontrolled explosion [41], but below the threshold, the current decreases together with stroke. For stable operation, the amplitude must be adjusted in a timely manner. If the current rises from cycle to cycle, $U_{p}$ must be reduced, but if it goes down, the amplitude must be increased. In Ref. [44], $U_{p}$ was adjusted manually, which did not allow fast tuning of the amplitude and led to unstable strokes.

**Figure 2:**
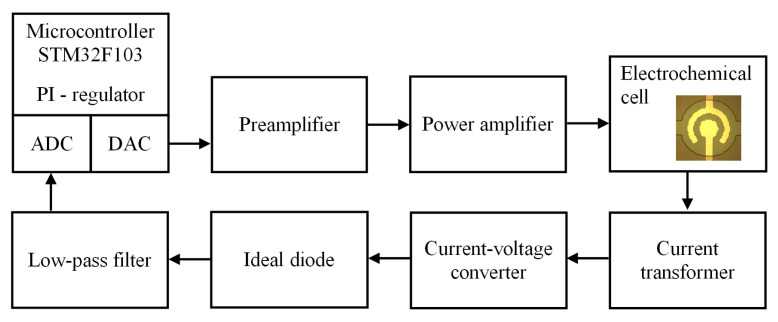
Operation scheme of the generator with a built-in current stabilization system.

In this work, we implemented a special technique to keep the performance stable. The adjustment of $U_{p}$ is carried out by a homemade pulse generator equipped with an automatic system of current tracking and keeping the current at a given level. The scheme of the generator is shown in Figure 2. The main unit is a microcontroller STM32F103. It supplies a series of pulses with the duration $t_{a}$ and sends it to a preamplifier via a digital-to-analog converter. The preamplifier shifts the DC component of the signal to create the AP pulses. These pulses are fed to a power amplifier with a voltage gain of 20. The amplifier provides a maximum output current of 1 A and an output voltage swing up to ±22 V. The amplified signal is applied to the electrochemical cell. The current flowing through the cell is reduced by 100 times by the current transformer and fed to the current–voltage converter based on an operational amplifier. The voltage that follows the shape of the current is applied to an ideal diode circuit, which converts the negative values to positive ones. Then, the signal is averaged by a low-pass filter based on the RC circuit. Thus, the voltage proportional to the average current $I_{a,i}$ during the *i*-th series of AP pulses is prepared and transferred to the analog-to-digital converter. The microcontroller compares $I_{a,i}$ with the target current $I_{0}$, which is preset by the user, and calculates an increment to the amplitude of the SP pulses using a proportional integral (PI) controller:

(1) $$\Delta U_{p,i}∼K\left(I_{0}-I_{a,i}\right),$$

where *K* is the PI coefficient, which takes a value from $0.5\times 10^{8}$ to $4.0\times 10^{8}$. Then, a series of SP pulses with the duration $t_{p}$ and the amplitude $U_{p,i}=U_{p,i-1}+\Delta U_{p,i}$ is applied to the electrodes, where $U_{p,i-1}$ is the amplitude of the previous series. After this, the cycle is repeated. Thus, the generator applies a series of AP pulses to the actuator, measures the average current, and compares it to the target value. The PI controller adjusts the amplitude of the SP pulses in such a way that the current approaches the target value in the next series. After a certain number of cycles, the current reaches the level to be maintained.

The membrane deflection is registered by a Michelson interferometer with orthogonal signals [39]. In order to increase the reflectivity, a 20 nm thick Al layer is deposited on the membrane. The voltage and current flowing through the electrodes are recorded by an oscilloscope, the PicoScope 5444D. The average current $I_{a}$ during the AP series and the power *P* consumed by the actuator per cycle are calculated from the waveforms of voltage U(t) and current I(t) as follows:

(2) $$I_{a}=\frac{1}{t_{a}}\int_{0}^{t_{a}}\left|I(t)\right|dt,$$

(3) $$P=f_{c}\int_{0}^{t_{a}+t_{p}}U\left(t\right)I\left(t\right)dt.$$

Oxidation of titanium in the current stabilization regime is studied for specially prepared samples. Al/Ti electrodes are formed on an oxidized silicon wafer in the same way as was done for the actuator, but no chamber is fabricated around the electrodes. The chip is placed in a Petri dish and filled with the electrolyte, where the electrochemical process is performed. The electrodes are inspected visually using an optical microscope equipped with a video camera, the Moticam 1SP. Their chemical composition is determined using an energy-dispersive X-ray (EDX) spectrometer, the Oxford Instruments INCA x-ACT, installed in a scanning electron microscope (SEM), the Zeiss Supra 40. The EDX analysis is performed at an accelerating voltage of 7 kV.

## 3. Results and Discussion

### 3.1. Restoring the Performance of Oxidized Electrodes

**Figure 3:**
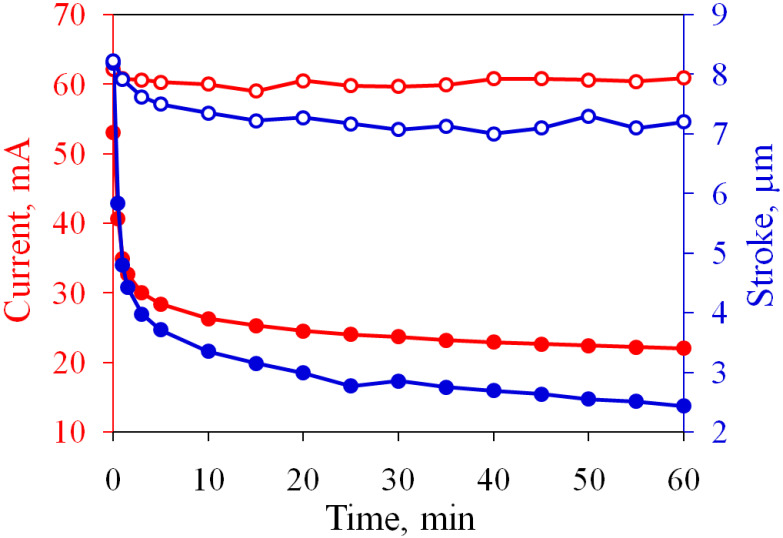
Time dependence of the average current and stroke. Filled and open circles correspond to the normal and new regimes, respectively.

Let us first demonstrate the proposed driving method in comparison with the normal working regime. We apply a series of AP pulses to a “fresh” actuator and drive it in the normal regime for an hour, while the SP pulses are not used during the passive time. The change in the average current and membrane deflection with time is shown in Figure 3. At the beginning of the test, the actuator conducts the current $I_{a}=53.1$ mA, which falls to 22.0 mA by the end of the test. The main drop in the current occurs during the first 5 min. The stroke exhibits similar behavior, since it is proportional to the volume of gas produced per series. This volume, in turn, is determined by the current flowing through the cell. At the beginning of the test, the actuator develops the stroke d=8.2μm, which is a typical value for a “fresh” sample. After 60 min, the stroke decreases to 2.4μm. Such a significant reduction is explained by the oxidation of the Ti working layer.

**Figure 4:**
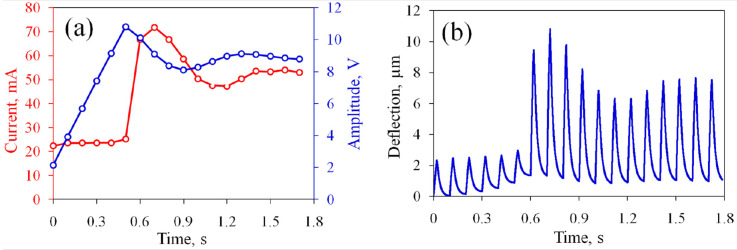
Operation of the actuator in the current stabilization regime during the first 18 cycles: (**a**) the average current and amplitude of the SP pulses; (**b**) the deflection of the membrane. The PI coefficient is $K=1.5\times 10^{8}$.

Next, the same device is tested in the current stabilization regime. The target current is set to be $I_{0}=55$ mA and slightly exceeds the initial value for the non-oxidized electrodes. The polarity of the SP pulses is positive and the PI coefficient is $K=1.5\times 10^{8}$. The current $I_{a}$ reaches the target value $I_{0}$ in several working cycles, as shown in Figure 4a. During the first AP series, the actuator conducts $I_{a}=22.3$ mA, which is close to the value observed at the end of the previous test. Since $I_{a}$ is significantly smaller than $I_{0}$, the generator immediately sets a non-zero amplitude $U_{p}=2.1$ V. This value does not provide the necessary increase in the current and $U_{p}$ increases further over the next five cycles with a step of $\Delta U_{p}\approx 1.7$ V and reaches 11.8 V. At the seventh series (t=0.6 s), the current rises sharply to 66.7 mA. This value exceeds the target level, so $U_{p}$ obtains a negative increment. After a few oscillations, the current gradually (in approximately 2 s) approaches $I_{0}$. The amplitude of the SP pulses also approaches a balance value of 9 V.

The membrane deflection during the stabilization process is shown in Figure 4b. At the first cycle, the membrane rises to 2.4μm and then returns to the zero position. However, during the subsequent cycles, the bottom position rises to 1.4μm at the sixth cycle. This means that the chamber does not completely eliminate the gas during the passive time. At the seventh cycle (t=0.6 s), the deflection increases sharply. This event coincides with the jump in $I_{a}$. In the following series, the upper position of the membrane reaches the maximum value 10.8μm and then stabilizes at 7.2μm.

**Figure 5:**
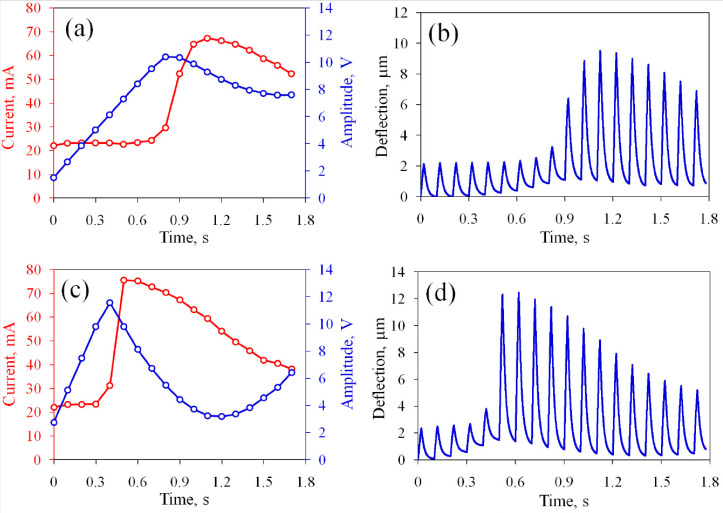
Time dependence of the current and amplitude of the SP pulses (**a**) and the membrane deflection (**b**) for $K=1.0\times 10^{8}$; (**c**,**d**) the same for $K=2.0\times 10^{8}$.

The sharp increase in the current during the stabilization process can increase the concentration of nanobubbles above the critical level and produce an explosion in the chamber [39]. Decreasing the PI coefficient allows the smoothing of the jump. The process at $K=1.0\times 10^{8}$ is shown in Figure 5a,b. One can see that the amplitude of the SP pulses and the current increase not so fast as for $K=1.5\times 10^{8}$. For larger *K* equal to $2.0\times 10^{8}$, the amplitude $U_{p}$ increases rapidly, providing a jump in the current to 75.6 mA and membrane deflection to 12.3μm, as shown in Figure 5c,d. Therefore, the larger value of *K* gives faster transition and stronger oscillation of the current. The range of *K* from $0.5\times 10^{8}$ to $1.0\times 10^{8}$ is optimal and we use $K=1.0\times 10^{8}$ in what follows.

The experiment demonstrates that the performance of the oxidized electrodes can be restored to the initial level. To investigate the long-term operation of the actuator, we drive it in the current stabilization regime during one hour. The target value is increased to $I_{0}=60$ mA in order to enhance the stroke. The time dependence of $I_{a}$ and *d* is shown in Figure 3 by open circles, where it can be compared with the normal driving regime. The current is kept well at a given level and varies from 59 to 62 mA, and the stroke is stabilized at d=7.2μm.

**Figure 6:**
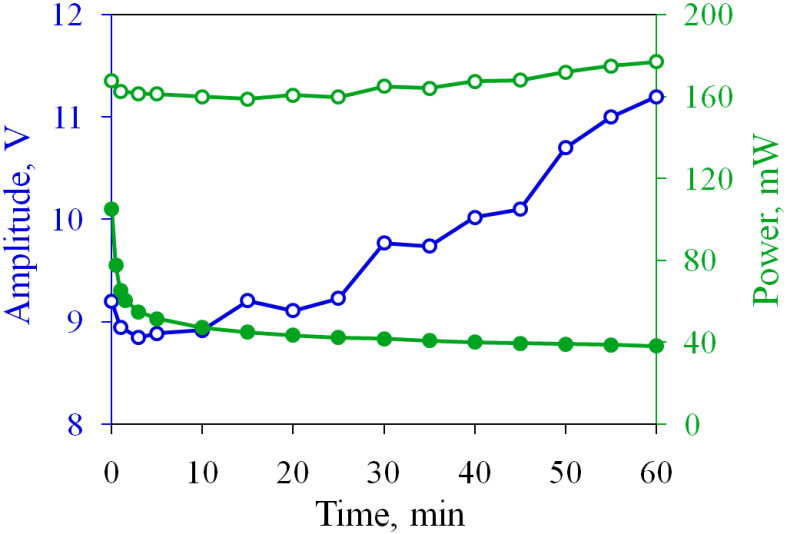
Time dependence of the amplitude of SP pulses and power consumption in the long-term test. Filled and open circles correspond to the normal and current stabilization regimes, respectively.

The change in the amplitude of the SP pulses with time is shown in Figure 6. In the first few minutes, $U_{p}$ decreases from 9.2 to 8.8 V, but then it demonstrates an upward trend. By the end of the test, the amplitude reaches 11.2 V. The power consumption of the actuator is also shown in Figure 6. The sample with the non-oxidized electrodes consumes P=105 mW. In an hour of operation in the normal regime, *P* is reduced to 38 mW due to the decrease in $I_{a}$. In the current stabilization regime, the power consumption increases, since the additional current flows through the electrodes during the passive time. At the beginning of the test, the actuator consumes P=168 mW, which is 60% higher than that for the “fresh” sample. The power increases to 177 mW in 60 min, which can be considered a relatively small increase.

### 3.2. The Actuator with Non-Oxidized Electrodes

**Figure 7:**
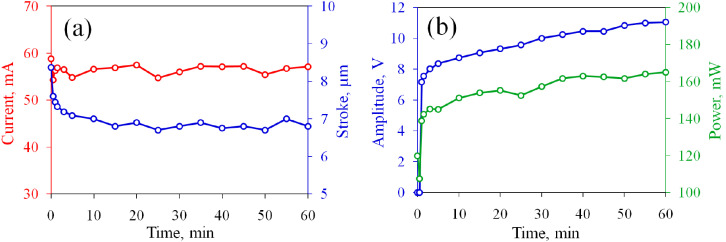
The actuator with non-oxidized electrodes in the current stabilization regime: (**a**) time dependence of the current and stroke; (**b**) the same for the amplitude of the SP pulses and consumed power.

As the next step, we implement the current stabilization regime in the actuator with non-oxidized electrodes. The target current is set as $I_{0}=57$ mA. The long-term operation is illustrated in Figure 7. At the beginning of the test, the electrodes conduct $I_{a}=59$ mA. This value is higher than $I_{0}$, so the SP pulses are not applied. However, the current decreases with time due to the oxidation and falls to 54 mA at the 30th second. Thus, the SP pulses are switched on at the first minute. Their amplitude rapidly increases to 7.2 V, but then it grows slower, with an average rate of 0.05 V/min. As in the previous experiment, $I_{a}$ deviates from $I_{0}$ by no more than 0.2 mA. The actuator generates a stroke of 8.4μm at the beginning of the test, which is reduced to 7.0μm and then stabilizes.

The power consumption is shown in Figure 7b. The starting power is equal to 120 mW, but then *P* quickly decreases to 108 mW due to the drop in $I_{a}$. Turning on the SP pulses at the first minute increases the power to 139 mW, i.e., by 16% compared to the initial value. By the end of the test, *P* increases by an additional 22% and reaches 165 mW. The final power and amplitude of the SP pulses are somewhat lower than those observed for the sample restored after the normal operation, but both actuators demonstrate similar working characteristics in the current stabilization regime.

It is interesting to estimate the performance of a hypothetical pump based on the fast actuator. If the stroke is maintained at d=7μm, the volume displaced by the membrane per cycle is estimated as $\Delta V=\pi r^{2}d/2\approx 0.7$ nL, where r=250μm is the radius of the chamber. A pump with ideal valves would provide the flow rate $R=\Delta Vf_{c}=0.41\;\mu$L/min. This is a high value for such a compact chamber. The overall volume pumped in 60 min would be 25μL. This volume corresponds to a reservoir with dimensions 5×5×1 mm, which is a reasonable capacity for an implantable drug delivery module. One hour of continuous operation is not a limit for the proposed actuator. The samples described in this section worked for two hours in the current stabilization regime. They did not show any visible degradation and the amplitude of the SP pulses continued to grow with the same rate of 0.05 V/min.

### 3.3. Oxidation of the Concentric Electrodes

**Figure 8:**
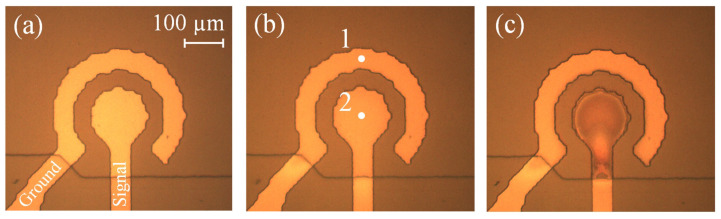
Photos of the concentric electrodes: (**a**) before the test; (**b**) after 60 min of operation in the normal regime; (**c**) after 60 min of operation in the current stabilization regime.

The chemical composition of the electrodes is investigated for the samples containing the electrodes without a chamber. The electrodes before the test are shown in Figure 8a. The contact lines are protected from the electrolyte by the SU-8 layer. First, the sample operates in the normal regime for an hour. The signal is applied to the inner electrode, while the outer one is grounded. At the beginning of the test, the sample conducts $I_{a}=60$ mA, but after 60 min, $I_{a}$ drops to 20 mA. In general, the current demonstrates the same behavior as observed for the actuator. The electrodes become darker in comparison with the original image, as one can see in panel (b). The darkening indicates the formation of the oxide layer on the surface of the electrodes [45]. The oxidation is confirmed by the EDX analysis data presented in Table 1. Oxygen is not detected at the “fresh” electrodes, but in 60 min, its content increases to 16.3 and 14.2% for the inner and outer structures, respectively. Both electrodes are oxidized because each of them alternately serves as a cathode and anode. The inner electrode exhibits a slightly higher amount of oxygen, since it has a smaller area and, therefore, a higher current density. The presence of aluminum and silicon in the signal is explained by the radiation coming from the conductive Al layer and from the Si substrate. The low amount of carbon is due to contaminations and residues of the photoresist.

Next, the same sample is tested in the current stabilization regime. The generator supports the current $I_{a}$ at 60 mA, while $U_{p}$ reaches 11 V in 60 min. The sample treated in this regime is shown in Figure 8c. The inner electrode darkens significantly, while the outer one practically does not change color in comparison with (b). The oxygen content of the inner structure increases up to 29.3% (see Table 1). Since the SP pulses are positive, this electrode acts as an anode during the passive time, which explains the strong oxidation. The outer electrode serves as a cathode and the amount of oxygen for this electrode decreases from 14.2 to 13.5% due to the reduction of titanium oxide. However, the significant recovery of the cathode is not observed.

**Figure 9:**
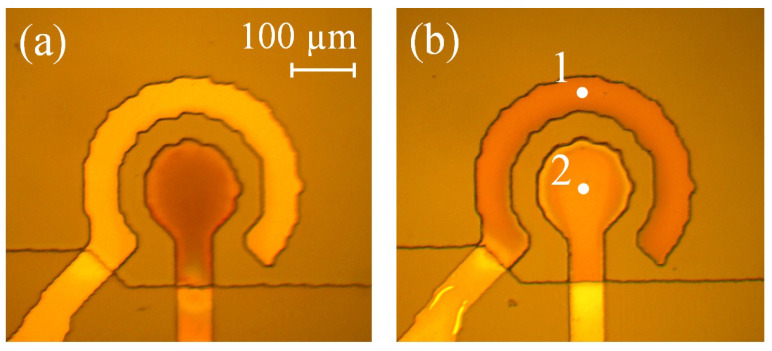
Concentric electrodes after the test with the positive (**a**) and negative (**b**) polarity of the SP pulses.

The oxidation of the “fresh” electrodes in the current stabilization regime is studied using two samples. For the first one, the SP pulses have a positive sign, while, for the other sample, the polarity is negative. In both cases, the current is maintained at 55–60 mA for 60 min. The electrodes after the test are shown in Figure 9 and the chemical composition is presented in Table 2. For the positive polarity, the inner electrode darkens strongly and demonstrates rather high oxygen content of 37.2%. The outer structure acts as a cathode during the passive time and looks much lighter. However, it exhibits 12.4% of oxygen, which is only 1.8% lower than the value observed after the normal operation. When the polarity is negative, both electrodes obtain a distinct brown color. The outer structure looks darker and contains 25.6% of oxygen, while the inner one exhibits 18.8%. In contrast to our expectations, both electrodes are oxidized more strongly than in the normal regime. Thus, the SP pulses suppress the oxidation of the cathode, but do not prevent it.

### 3.4. Oxidation of the Rectangular Electrodes

**Figure 10:**
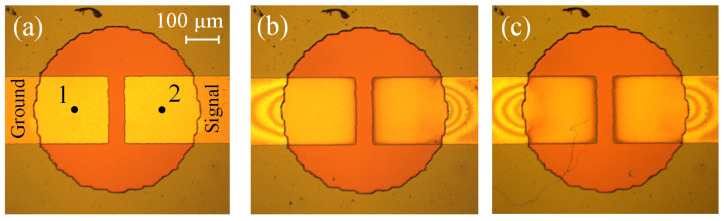
The rectangular electrodes: (**a**) before the test; (**b**) after 60 min of operation in the current stabilization regime with the positive polarity of SP pulses; (**c**) after additional 60 min with the negative polarity pulses.

Concentric electrodes may provide incorrect information about the oxidation in the current stabilization regime due to their asymmetry. The inner electrode has a smaller area and operates at a higher current density than the outer one. To avoid this effect, we fabricate symmetric rectangular structures, as shown in Figure 10a. First, the rectangular electrodes are tested in the normal regime for an hour. The signal is applied to the right electrode, while the left one is grounded. During the test, $I_{a}$ drops from 49 to 28 mA. This decrease is not as prominent as that observed for the concentric electrodes, since the rectangular structures provide approximately two-times lower current density and are oxidized more slowly [44]. After the test, the left and right electrode exhibit 8.8 and 8.6% of oxygen. Thus, both electrodes are oxidized equally, which is accompanied by a slight darkening.

**Figure 11:**
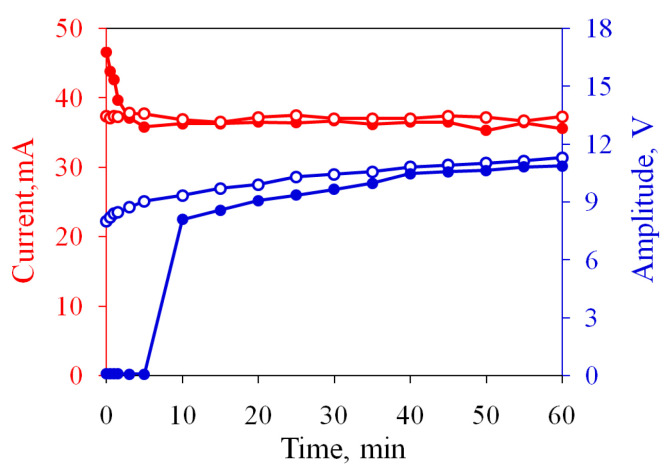
Time dependence of the current and amplitude of the SP pulses for the rectangular electrodes. Filled and open circles refer to the positive and negative polarity of the SP pulses, respectively.

Next, we use a “fresh” sample in the current stabilization regime with the positive SP pulses and then change the polarity to negative. In both cases, the current is kept at 36–37 mA for 60 min. In contrast to the concentric electrodes, both tests are performed with the same sample. The current and amplitude of the SP pulses are demonstrated in Figure 11. For the positive polarity, the SP pulses are switched on at the 10th minute. Their amplitude increases from 8.1 to 10.9 V during the test. As one can see in Figure 10b, the electrodes darken in comparison with the original image in Figure 10a. The right electrode darkens more strongly, which is especially noticeable at the edges. This reflects the oxygen content of 13.5%; see Table 3. The left electrode contains 7.7% of oxygen, which is only 1.1% lower than the amount of O after the normal operation. One can conclude that the current stabilizing regime provides stronger oxidation compared to the normal regime, which confirms the conclusions drawn for the concentric electrodes.

After application of the positive pulses, the same sample is tested for the negative polarity of the SP pulses. To keep the current fixed, one has to apply the SP pulses from the very beginning. The amplitude starts from 8.0 V and demonstrates a rapid increase during the first 5 min, but then the growth slows down, as one can see in Figure 11. The sample after the test is shown in Figure 10c. The left electrode darkens compared to the previous image because it acts as an anode. The oxygen content increases from 7.7 to 13.8%; see Table 3. The right electrode practically does not change in color, but the oxygen percentage decreases from 13.5 to 12.2%. Again, we can conclude that the new regime reduces titanium at the cathode only partially.

In general, the experiments with rectangular electrodes confirm the conclusions drawn for the concentric structures. The use of the SP pulses for the non-oxidized electrodes does not prevent the oxidation of Ti at the cathode, but only suppresses it. If the current stabilization regime is applied to pre-oxidized electrodes, a significant reduction at the cathode does not occur, although the current is maintained at the level demonstrated by the non-oxidized structures. In both cases, the SP pulses intensify the oxidation of Ti at the anode. As a result, the total amount of oxygen in the electrodes increases compared to the normal regime. The growth of the oxide layer on the anode raises the resistance of the electrochemical cell. This is the reason that maintaining the current requires a gradual increase in $U_{p}$.

Since the SP pulses do not provide significant reduction of the preliminarily oxidized cathode, but the current corresponds to the non-oxidized sample, it is natural to assume that the surface layer on the electrode plays an important role. The SP pulses modify this layer in such a way that it is able to support a high current during the subsequent AP series. Local reduction of the titanium oxide may occur or cracks can appear in the oxide layer, which are sufficient to maintain the current during the active time.

### 3.5. Damage of the Electrodes

**Figure 12:**
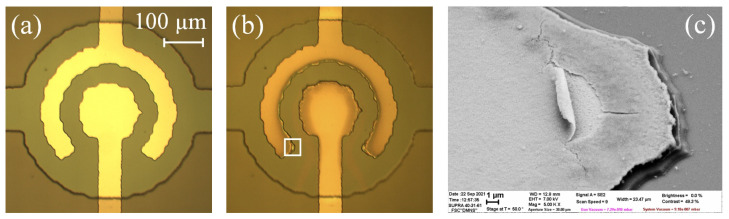
Working chamber of the actuator with the transparent membrane: (**a**) before the test; (**b**) after 60 min of operation in the current stabilization regime; (**c**) SEM image of the electrode edge marked by the white frame in image (**b**).

The current stabilization regime ensures the long-term operation of the actuator, with a large stroke corresponding to the intensive production of nanobubbles inside the working chamber. The nanobubbles containing a mixture of H${}_{2}$ and O${}_{2}$ gases combust near the electrode and provide strong mechanical action on the surface. This phenomenon leads to erosion of the material, as was recently demonstrated for platinum [42]. To observe the damage to the Al/Ti electrodes, we used actuators without a reflective Al layer at the membrane (the layer is used to observe the membrane movement interferometrically). The sample worked for 60 min at $I_{a}$ = 55–59 mA. The chamber before and after the test is shown in Figure 12a,b. The electrodes turned brown due to the oxidation. Their edges darkened especially strongly, since the current density in these regions was the highest.

The corners of the outer electrode demonstrated an atypical change in color. SEM inspection revealed the damage to these areas. The surface layer cracked and peeled away from the electrode, as shown in Figure 12c. The delaminated layer had a thickness of around 100 nm. A probable reason for this phenomenon is the mechanical stress generated in the titanium oxide film during its growth. The rolled shape of the exfoliated layer indicates the stress gradient. The formation of the stress during the anodic oxidation of Ti was described in several papers [46,47,48,49,50]. In general, the structures were damaged not very significantly. The removal of the material under the mechanical action of nanobubbles was not observed. This can be explained by the relatively high hardness of Ti (Brinell hardness is 716 MPa), which is considerably higher than that for Pt (392 MPa). Titanium oxide grown by the electrochemical oxidation technique is 1.8–3.5 times harder than pure Ti [51,52] and it can protect the electrodes from destruction.

## 4. Conclusions

A new driving regime of the fast electrochemical actuator has been demonstrated in which the time interval between the series of AP pulses is filled with SP pulses. This regime was proposed earlier [44] but was not implemented due to an inability to adjust the amplitude of the SP pulses. In this work, the amplitude has been corrected automatically using a homemade generator equipped with a PI controller. It keeps the current through the chamber constant, supporting the membrane deflection at 7–8 μm during at least an hour of continuous operation at 10 Hz. The current reaches the target level in several working cycles and the transition process is controlled by the PI coefficient. The use of the SP pulses increases the power consumption by 15–60% depending on the oxidation state of the electrodes. This is due to an additional current flowing through the electrodes during the passive time, which also is responsible for the slightly lifted position of the membrane. A gradual increase in the amplitude of the SP pulses with an average rate of 0.05 V/min is necessary to maintain the high performance of the actuator.

Oxidation of the electrodes in the new regime has been investigated using concentric and rectangular structures. The current stabilization regime slightly reduces the oxidation of the cathode, while the anode is even more oxidized. The growth of the oxide layer raises the resistance of the electrochemical cell. This is the reason for the increase in the amplitude of the SP pulses during the operation. Mechanical stress generated in the titanium oxide film causes minor cracking and delaminating of the surface layer of the electrodes but does not disturb the operation of the actuator.

## Figures and Tables

**Table 1:** Chemical composition of the concentric electrodes before and after the test in the normal and new regimes. EDX analysis is performed at the regions marked by the white points in Figure 8b. The content of chemical elements is given in atomic percent.

Chemical Element	Before the Test		After the Test, Normal Regime		After the Test, New Regime	
	Region 1	Region 2	Region 1	Region 2	Region 1	Region 2
C	1.7	1.9	1.0	0.8	-	0.8
O	-	-	14.2	16.3	13.5	29.3
Al	0.8	0.8	0.8	0.7	0.8	0.7
Si	0.4	0.3	0.6	0.5	0.4	0.4
Ti	97.0	97.0	83.4	81.7	86.0	68.9

**Table 2:** Chemical composition of the concentric electrodes after the test with the positive and negative polarity of SP pulses. EDX analysis is performed at the regions marked by the white points in Figure 9b.

Chemical Element	Positive Polarity		Negative Polarity	
	Region 1	Region 2	Region 1	Region 2
C	0.6	2.0	-	0.5
O	12.4	37.2	25.6	18.8
Al	0.7	0.6	0.7	0.7
Si	0.4	0.3	0.3	0.3
Ti	85.9	59.9	73.4	79.7

**Table 3:** Chemical composition of the rectangular electrodes before and after the test in the new regime with the positive and negative polarity of SP pulses. EDX analysis is performed at the regions marked by the black points in Figure 10a.

Chemical Element	Before the Test		After the Test, Positive Polarity		After the Test, Negative Polarity	
	Region 1	Region 2	Region 1	Region 2	Region 1	Region 2
C	-	-	-	-	-	-
O	-	-	7.7	13.5	13.8	12.2
Al	0.8	0.7	0.7	0.7	0.9	0.8
Si	0.4	0.2	0.1	0.3	0.4	0.3
Ti	98.8	99.1	91.5	85.4	85.0	86.7

## Data Availability

Not applicable.
